# Investigation on Hydration and Mechanical Properties of Mortar Containing Limestone Powder and Fly Ash Based on the Coupled Chemical–Thermal–Mechanical Method

**DOI:** 10.3390/ma13194387

**Published:** 2020-10-01

**Authors:** Wei Zhou, Wenxiang Tian, Tianqi Qi, Shuhua Liu, Chuqiao Feng, Gang Ma, Xiaolin Chang

**Affiliations:** 1 State Key Laboratory of Water Resources and Hydropower Engineering Science, Wuhan University, Wuhan 430072, China; zw_mxx@whu.edu.cn (W.Z.); tian_wenxiang@whu.edu.cn (W.T.); tianqi_qi@whu.edu.cn (T.Q.); shliu@whu.edu.cn (S.L.); magang630@whu.edu.cn (G.M.); 2 Guizhou Survey/Design Research Institute for Water Resources and Hydropower, Guiyang 550002, China; fcq0418@163.com

**Keywords:** composite cement-based material, chemical–thermal–mechanical method, random pore model, hydration characteristics, mechanical property evolution

## Abstract

The composited cementitious materials usually have superior performance; for example, using limestone powder (LP) and fly ash (FA) as the admixtures of cement in concrete/mortar is a popular way of improving the properties of concrete/mortar structures. In this work, we performed experimental tests to study the hydration process and pore distribution in mortar containing different ratios of LP and FA. Based on the results of mercury intrusion porosimetry (MIP), a numerical mortar model with random pore is built. The model can reflect the synergistic hydration interaction and filling effect caused by the admixtures of LP and FA. After analyzing the hydration process, the coupled chemical–thermal–mechanical method was used to simulate the characteristics of mortar containing LP and FA. The coupling model can simulate the “hump-type” hydration acceleration stage of the mortar at early age, which is specifically caused by the LP, proved in the experimental test. Additionally, the special, “hump-type” stage is important to enhance the early strength of the mortar. At different levels of admixture content, the random pore model and coupled method can predict the evolution process of the mechanical properties well, at early age and for long-term strength. Both experimental and numerical results suggest that the mortar containing admixtures of the proper ratio of LP to FA have good mechanical properties, which can be applied to engineering structures.

## 1. Introduction

Portland cement has been widely used in hydraulic engineering, transportation, industrial, and civil construction for many years [1]. To improve the durability and mechanical properties of concrete, many kinds of admixtures are added to the cement matrix, such as fly ash, silica fume, limestone powder, and slag powder. For mass concrete structure, high-fly-ash-content concrete can solve the problem of excessive increases in temperature in the hydration process [2]. However, with the construction of massive concrete structures such as dams, the resources of fly ash tend to be scarce. Using limestone powder to replace fly ash as a cement-based admixture can significantly solve the fly ash shortage problem. In addition, the admixture of limestone powder with high quality [3,4,5] and low cost can reduce the cost of construction and transportation, and also solve environmental problems.

The concrete containing limestone powder (LP) and fly ash (FA) has been widely tested and analyzed in previous studies. The effects on strength, stability, and durability were gradually discovered. Kirk Vance et al. [6] and V. Bonavetti et al. [7] found that the filler effect of LP could accelerate the early hydration of cement. K. De Weerdt et al. [8,9] verified the synergetic interaction between LP and FA; that is, the ettringite was prevented from decomposing into unstable monomer sulfate. Additionally, limestone powder can improve the grain packing of cement and the dispersal of clinker particles, as demonstrated by E.F. Irassar et al. [10]. Tang et al. [11] found that LP could contribute to dispersing the pore structure of blended cement paste. For mechanical properties, Menendez G et al. [12] and V. Bonavetti et al. [13] verified that the early strength of concrete could be enhanced by adding LP. The previous research indicated that there are many advantages of adding LP and FA to concrete compared with using only ordinary Portland cement (OPC), especially for its higher early strength and stable hydration products. Usually, the content of admixtures is from 5% to 30%, but there is some research on high content. This article describes the effect of increasing the contents of LP and FA in concrete. Here, total content is up to 50%.

The strength evolution of concrete is closely related to the hydration of cement-based materials. For an experimental test, the mechanical properties of concrete can be easily obtained through a destructive test, but it is hard to predict the strength via an undamaged test. Therefore, numerical simulation is ideal for predicting strength evolution, especially when considering the hydration process of the cement paste [14,15]. To simulate the evolution of the hydration and the strength characteristics, the coupled chemical–thermal–mechanical method is applied to the mesoscale mortar model. The multi-field coupling method is based on the hydration kinetics of cement paste that has been used since the 1990s. The hydration model of Arrhenius included in this method is widely accepted [16,17,18]. Cervera et al. [19] proposed a hydration degree model to simulate the chemical–thermal–mechanical coupling behavior of concrete, which can be implemented and predicted in the finite element method. Many researchers have since used this coupling model to simulate mass concrete [20] and mesoscale concrete. Gawin et al. [21] proposed another polynomial formula to describe the affinity of the hydration reaction. Zhou et al. [22,23,24] modified the hydration degree model proposed by Cervera and Gawin and applied it to analyze mesoscale concrete. In the chemical–mechanical coupling part, Bentz D P et al. [25] and Ulm F et al. [26] linked the mechanical property evolution with the hydration degree. Mazars et al. [27] proposed the concept of the damage factor, which can describe the change in concrete bearing capacity. All these lay a foundation for the damage evolution model of concrete based on the coupled chemical–thermal–mechanical method.

Concrete is regarded as a typical porous material, whose strength depends much on the hole due to the weakness of the pores. For the concrete containing LP and FA, LP has a filling effect that changes the porosity, which also directly affects the mechanical property. To simulate the evolution of the mechanical property accurately, the pore structure should not be neglected in meso-modeling [28]. Pores are usually set in solid models to simulate the porous material, such as the random pore model. However, the mechanical parameters of the composite phase are easily measured in concrete, such as the external strength and elastic modulus, but the parameters in each single phase inside are difficult to obtain. Many researchers have established the relationship of the parameters between the composite phase and the single phase, such as the solid and the pore in porous materials. Kemeny, J. et al. [29], Tang [30], and other researchers, who applied an equivalent relationship to model porous materials such as concrete, proposed the method about the equivalent elastic modulus of porous materials.

This paper aims to investigate concrete containing high amounts of LP and FA, including their hydration characteristics, macro-mechanical properties, micro-hydration products, and pore size distribution. In addition, a multi-field coupling method that can reflect the hydration characteristics of composite cementitious materials is developed, which can consider the effect of different ratios of LP and FA in the mesoscale mortar. The model can also simulate the evolution of strength and fracture characteristics under uniaxial compression at different hydration ages of the mortar.

## 2. Materials and Experiment

### 2.1. Materials

The cement-based materials in the mortar were ordinary Portland cement (OPC), fly ash (FA), and limestone powder (LP). The strength grade of OPC is 42.5 MPa, the fly ash conformed to the stair FA property of the Chinese National Standard DL/T5055-2007, and the limestone powder was from carboniferous limestone, which was ground for 50 min by a vertical grinder. The chemical compositions of these three components determined by X-ray fluorescence (XRF) are listed in Table 1. It can be seen that the mass percentage of CaO and loss on ignition was about 95%; the loss on ignition was mainly from the CO_2_ produced by decomposition, which proves that the limestone powder contains a high purity of 95% CaCO_3_ content.

### 2.2. Experimental Method

#### 2.2.1. Mix Proportions

To study the characteristics of mortar containing a high content of admixtures, the mortar consisted of composite cement and was designed with different ratios of LP and FA. The total admixture content was kept at 50% with the water–binder ratio setting at the same value of 0.5. The mortar mixture proportions are shown in Table 2. For each proportion, the mortar specimens (40 mm × 40 mm × 160 mm referred size in the Chinese National Standard GB/T 17671-1999) were cast from a blend of 225 g of OPC, 225 g of admixture, 1350 g of normalized sand, and 225 g of water. The fluidity of the mortar is listed in the last column of Table 2. Because of the high water-binder ratio of the mortar, no superplasticizer was added in the mix proportion. With the rising content of LP, the fluidity increased.

#### 2.2.2. Test Methods

To obtain the hydration heat characteristics of composite cement-based materials containing different ratios of LP and FA, the heat flow of the mortar samples was monitored using a TAM Air isothermal calorimeter at 25 °C during the initial 72 h of the hydration process. The TAM Air had eight parallel twin-chamber measuring channels maintained at a constant temperature: one chamber containing the sample, and the other containing the reference, always water. The temperature fluctuation was less than ±0.02 °C, and the precision could reach ±20 mµW. The water-to-binder ratios (W/B) were 0.5 and 5 g for each sample used. First, 5 g of powder and 2.5 g of water were weighed for each specimen as the testing reference, and 2.90 g of water were used as the control reference. The testing reference was mixed, and the testing and control references were placed into certain tunnels at the same time. Then, marking the data till the equipment became stable.

The compressive strength and flexural strength of the mortar was tested following a method in the Chinese National Standard GB/T 17671-1999 (Method of Testing Cement-Determination of Strength) after curing the specimens at a temperature of 20 ± 1 °C and a relative humidity of ≥90%, until the stipulated age of 7, 28, 90, 180, and 365 days. The strength was tested by a YAW-300C, a battery solution type flexural and compressive testing machine. The flexural strength was calculated from the average value of the three prism specimens of 40 × 40 × 160 mm. The compressive strength was calculated from the average value of six cubic specimens testing results.

After the mechanical test of the mortar was performed at each stipulated curing age, the inner crushed cement slurry powder of the mortar was collected and soaked in anhydrous ethanol to terminate the hydration, and the microscopic characteristics test was performed.

Mercury intrusion porosimetry (MIP) was used to test the pore distribution of the hydrated composite cement matrix with three different ratios (LF-0/LF-30/LF-50). Mung-bean-like crushed powder was prepared for this experiment. The prepared samples were immersed in anhydrous ethanol and dried in a 60 °C desiccators for 24 h. After that, each sample (about 1.5 g) was put into the glass tube of a dilatometer for a low pressure test, and the glass measuring tube filled with mercury was then placed into a high-pressure measuring tank for a high pressure test. The mercury injection apparatus was the Auto Pore IV (9500). The pressure ranged from 0.20 to 60000.00 psi (about 1.38 kPa to 413.76 MPa), and calculation of the measuring pore size range was from about 30 Å to 0.9 mm.

The rest of the sample inner pieces were ground in the agate mortar and dried at 60 °C for 2 h to reduce carbonization for X-ray diffraction (XRD). Here, the XRD test was used to identify the hydration products of the cement paste during the hydration process, in order to find out the effect of the limestone powder on the microstructure in the composite cementitious materials.

### 2.3. Experimental Results

#### 2.3.1. Compressive and Flexural Strength

**Figure 1:**
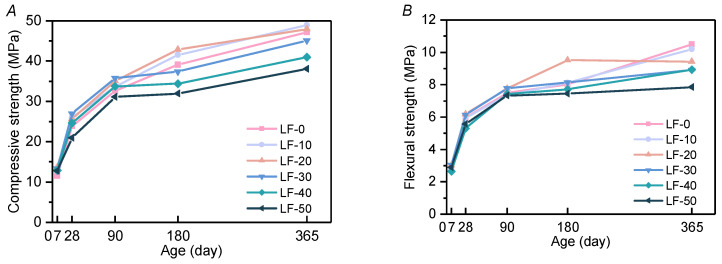
Compressive and flexural strength of all samples at 7/28/90/180/365 days. (**A**) Compressive strength; (**B**) flexural strength.

The compressive and flexural strength of the mortar is shown in Figure 1. For the early-age strength of all samples, before 28 days, compressive strength and flexural strength, when increasing limestone powder content, showed a trend of first increasing and then decreasing, which is consistent with previous studies from other composite cementitious materials mixed with limestone powder [31,32]. For the final strength of all samples, both compressive strength and flexural strength varied little when the substitution content of limestone powder was lower. The cement-based material of fly ash was excessively replaced by limestone powder, which will sharply decrease the strength later. This is because limestone powder accelerates the hydration of cement at an early age, promoting the growth of early strength, but too much limestone powder will dilute the strength provided by cement. Additionally, the dilution effect can affect the pozzolanic reaction of fly ash at a later age [33], cause a decrease in final strength. As shown by the slope from 28 days to 365 days in Figure 1, the compressive strength increased largely from 28 days to 365 days in LF-0, LF-10, LF-20, while this strength growth was not so fast corresponding to the low fly ash content of LF-40 and LF-50. However, the appropriate proportion of LP and FA will cause a synergetic interaction, which makes the strength of the mortar increase temporarily at 180 days, as shown by LF-10 and LF-20. The same phenomenon can also be seen in other ternary cementitious materials [34,35].

#### 2.3.2. Isothermal Calorimeter

**Figure 2:**
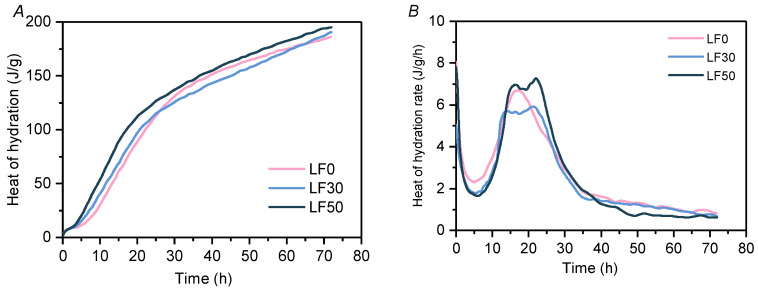
Heat of hydration and heat of hydration rate in LF-0, LF-30 and LF-50 mortar matrix. (**A**) Heat of hydration, (**B**) heat of hydration rate.

In order to study the role of limestone powder in the hydration process of cement slurry, the heat of hydration and the hydration rate of the blend paste were analyzed by the isothermal calorimeter. The tested samples include LF-0, LF-30, and LF-50. Figure 2A shows the release curve of the heat of hydration, and Figure 2B shows the heat of hydration rate curve. As shown in Figure 2B, the slope of the first peak of LF-30 and LF-50 after the induction period is larger than that of LF-0, which is also reflected in Figure 2A; that is, the hydration heat release of LF-50 is greater than that of the other two samples. Based on the hydration kinetic model proposed by G. De Schutter [18], the hydration process of LF-0, LF-30, and LF-50 was analyzed. The leading process of LF-0 and LF-30 was NG-I-D, whereas LF-50 was NG-D; the “I” stage was missing. Thus, high limestone powder content can change the progress of the hydration kinetics, which can enhance the hydration of cement and provide more nucleation sites for C-S-H [3]. The heat of hydration rate peak was lower in the LF-30 mortar matrix than it was in LF-0 and LF-50. This is because the limestone powder can dilute but not participate in the hydration reaction between fly ash with cement at an early age. In addition, it is obvious that there is a hump-type hydration curve in the limestone-doped cementitious materials (LF-50 and LF-30 compared with LF-0). The first peak of the “hump-type” stage may be mainly caused by the nucleation effect of the limestone powder. Martin et al. [36] and other researchers [37,38] confirmed that the acceleration effect of composite cement containing limestone powder is also promoted by the nucleation effect. The second peak of the “hump-type” stage occurred in LF-30 and LF-50, which can be attributed to the participation of the aluminate phases reflecting AFm formation. The same phenomenon has also been discovered in previous researches [39,40,41].

#### 2.3.3. Hydration Products

**Figure 3:**
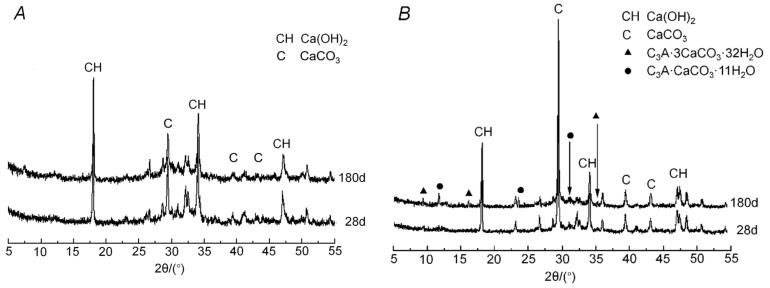
XRD analysis of OPC and cement matrix of LF-30. (**A**) OPC, (**B**) LF-30.

To understand the synergistic hydration interaction of LP and FA, we compared hydration products with pure cement and those with composite cement containing LP and FA. The hydrated cement paste powder of the OPC and LF-30 were tested by XRD at hydration ages of 28 and 180 days. The XRD results are shown in Figure 3. Compared with the main diffraction peaks of these two samples, the main hydration production is Ca(OH)_2_. In Figure 3A some diffraction peaks were not marked out because of the existent of anhydrous cement particles, hence the production of CaCO_3_ was mainly caused by carbonation. A synergistic interaction of LP and FA was observed when studying the additional reaction of aluminates. The interaction product was calcium carboaluminate hydrate [8]. The same hydration products of calcium aluminate tricalcium carbonate and calcium aluminate monocarbohydrate could be observed in the XRD of LF-30, proving that the limestone powder participated in the hydration reaction. Therefore, the proper addition of limestone powder can produce stable products and will not significantly reduce strength in a later hydration stage.

#### 2.3.4. Pore Structure

**Figure 4:**
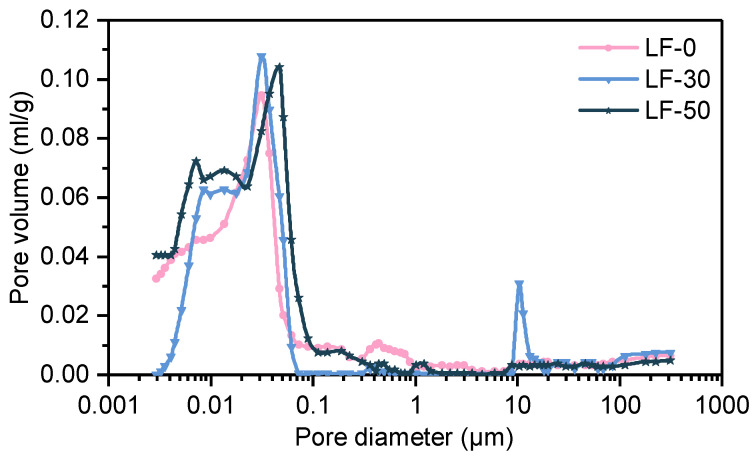
Pore distribution of cement matrix in LF-0, LF-30, and LF-50.

Because of its convenience and repeatability, MIP test is widely used in comparing the pore structure characteristics of different proportions of cement-based materials [42,43]. Although there is an argument that the MIP test may be unsuitable to get the real particle size distribution (PSD) of cement-based materials [44,45] because of the existence of the ink-bottles type pores. The MIP test was carried out on a hydrated blend paste of samples LF-0, LF-30, and LF-50. Their total porosities and pore distributions were measured [43]. Figure 4 shows the analysis result of pore distribution. It can be seen that pores smaller than 0.02 μm (d (diameter)) were obviously increased, and the pores larger than 0.1 μm were reduced in LF-30 and LF-50, which proves that limestone powder can significantly reduce large pores in mortar. Based on the theory of pore classification put forward by Wu Zhongwei, four grades of porosity were classified, as shown in Table 3. The three groups of mortar samples (LF-0, LF-30, and LF-50) all contained 50% auxiliary cementitious materials. Their porosities were in the following order from small to large: LF-30, LF-0, and LF-50. The porosity of the mortar with 50% fly ash was slightly lower than that with 50% limestone powder, which is mainly due to the secondary hydration of the fly ash in later stages [46]. When LP and FA were mixed, the porosity of the mortar was the lowest, which indicates that the synergistic reaction of the filling effect by LP and the pozzolanic effect by FA could effectively improve the microstructure of the mortar. The lowest pore distribution at each range is caused by different samples. Regarding harmful pore sizes (d > 0.2 μm), the proportion of harmful pores in the limestone mortar was significantly lower than that in the fly ash mortar. Generally, adding LP and FA has a good filling effect. The paste is more compact, and the porosity of the mortar is reduced, thus improving its pore size distribution. However, the effect of LP usually depends on its particle size distribution (PSD). Some scholars find that LP can enlarge the average pore size [3,47], whereas others found that it has a positive effect [48]. Pore classification will serve as a basis for random pore generation in the numerical simulation in the next section.

## 3. Simulation Method

In the engineering structure, although the mechanical properties of concrete/mortar can be obtained through physical experiments, it can take as many as several months or even a year. It is essential to use an appropriate method to predict the properties of concrete/mortar through a numerical test. Because of the variation in hydration properties of complex cement paste, the process of the strength evolution of the concrete/mortar cannot be neglected. Here, an effective method called the chemical–thermal–mechanical method and the random pore model is used to model the mechanical evolution process of mortar, according to the information of aggregate grade, cement hydration, and porosity.

### 3.1. Chemical–Thermal–Mechanical Method

In concrete/mortar, the exothermic reaction of the cement paste is due to the water combination, which can release heat known as the heat of hydration. The degree of hydration is defined as the heat released during hydration to the ultimate released heat when the hydration is complete. It can be formulated as follows [18,49]:

(1) $$\xi \left(t\right)=Q\left(t\right)/Q_{\infty }$$

where Q(t) is the accumulative heat of hydration at time t, $Q_{\infty }$ is the final volumetric heat of hydration at the ultimate time, and ξ(t) is the degree of hydration at time t.

The hydration rate is related to the chemical affinity and reaction temperature according to Arrhenius’s law [50], shown as Equation (2):

(2) $$\partial \xi /\partial t=A_{\xi }\left(\xi \right)\cdot \exp\left(-E_{a}/RT\right)$$

where $A_{\xi }\left(\xi \right)$ is the chemical affinity that is temperature-dependent, $E_{a}$ is the reaction activation energy, T is the reaction temperature, and R is the ideal gas constant.

Cervera and Oliver [19] divided the free energy of the chemical-thermal system into three contributions: the thermal, the chemical, and the chemical-thermal coupling contribution. They assumed that the chemical contribution is a cubic function of the degree of hydration, so the expression of the chemical affinity can be derived using the strategy presented in their research. Feng et al. [22] optimized this assumption and assumed that the chemical contribution is a quaternary function of the degree of hydration. The function of an analytical form of the normalized affinity based on thermodynamics is derived by using the same strategy as Cervera et al. [19], as follows:

(3) $$A_{\xi }\left(\xi \right)=\beta_{1}\left(\beta_{2}+\beta_{3}\xi +\xi^{2}\right)\left(\xi_{\infty }-\xi \right)\cdot \exp\left(-\bar{\eta }\xi /\xi_{\infty }\right)$$

where $\beta_{1}$, $\beta_{2}$, and $\beta_{3}$ are the material coefficients, $\xi_{\infty }$ is the ultimate degree of hydration, which is often set as 1, and $\bar{\eta }$ represents the viscosity due to micro-diffusion of the free water through the already formed hydrates. These parameters can be calibrated through the experimental results.

**Figure 5:**
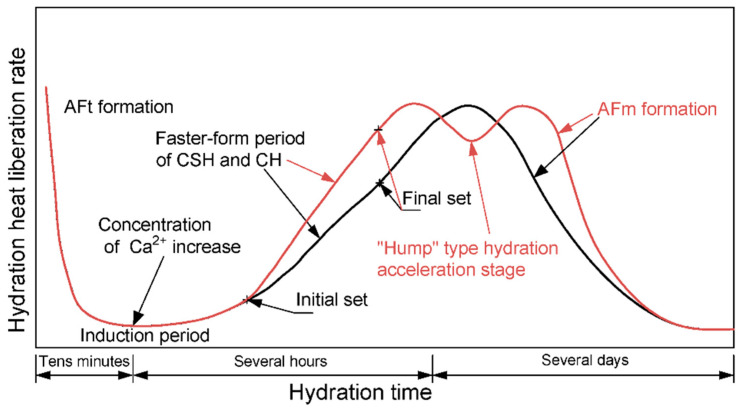
Schematic diagram of the hydration heat liberation rate for ordinary Portland cement (the black curve) and composite cement mixed with limestone powder (red curve).

For OPC, the hydration heat liberation rate is a single peak. The whole hydration process can be divided into periods of initial reaction, slow reaction, acceleration, and deceleration [51], which are represented by the black curve in Figure 5 [52]. However, for composite cement containing LP and FA, a “hump-type” hydration acceleration stage can be widely discovered in serial experiments [46,53,54,55], which is generally considered to be caused by the generation of ettringite (AFm formation) [39,40]. The red curve in Figure 5 shows a schematic diagram of the hydration heat liberation rate for composite cement mixed with limestone powder.

To simulate the “hump phenomenon” of the heat of hydration rate in composite cementitious materials, the additional “hump” hydration function model is put forward as follows:

(4) $$A_{Hump}\left(\xi_{2}\right)=\exp\left(b-\xi_{2}{}^{2}/a\right)\cdot \xi_{2}{}^{4}$$

where $\xi_{2}$ is the adjusted degree of hydration that can relate to the degree of hydration of the OPC. a and b are the coefficients that can control the range of the “hump” hydration function. The following equation can calculate the $\xi_{2}$:

(5) $$\xi_{2}=\left(2\xi -\left(\xi_{valley2}+\xi_{valley1}\right)\right)/\left(2\left(\xi_{valley2}-\xi_{valley1}\right)\right)$$

where $\xi_{valley1}$ is the degree of hydration at the initial set of the faster-form period of CSH and CH in OPC, and $\xi_{valley2}$ is the degree of hydration at the end of AFm formation. The two parameters should be obtained based on the characteristics of the “hump-type” stage such as the initial set shown in Figure 5. For other binder paste with different compositions, they should be recalibrated. 

The function of an analytical form of the normalized affinity of composite cement containing LP and FA can be derived. Equation (6) is the superposition of Equations (3) and (4), in which Equation (3) can demonstrate the hydration of the OPC, and Equation (4) can demonstrate the hydration of the FA and LP:

(6) $$A_{\xi }\left(\xi \right)=\beta_{1}\left(\beta_{2}+\beta_{3}\xi +\xi^{2}\right)\left(\xi_{\infty }-\xi \right)\cdot \exp\left(-\bar{\eta }\xi /\xi_{\infty }\right)+\exp\left(b-\xi_{2}{}^{2}/a\right)\cdot \xi_{2}{}^{4}$$

The transient heat transfer process of the mortar can be described as follows:

(7) $$\rho C\cdot \partial T\left(x,y,z,t\right)/\partial t=\lambda_{T}\Delta T\left(x,y,z,t\right)+\partial Q/\partial t$$

(8) $$\partial Q/\partial t=Q_{\infty }\cdot \partial \xi /\partial t$$

where ρ is density, C is volumetric heat capacity, $\lambda_{T}$ is thermal conductivity coefficient, $Q_{\infty }$ is the final volumetric heat of hydration, and Δ is Laplacian operator. The latent heat release is a nonlinear and thermally dependent process because of the complex mortar hydration.

In the process of iteration, the rate of hydration degree was calculated based on the Equations (2) and (6), according to the hydration degree and temperature at previous moment. The hydration degree ξ at next moment was obtained by integration. Then, the heat of hydration and heat of hydration rate can be calculated by Equations (1) and (8). 

The damage and failure process of mortar can be simulated by a damage model, which can be described by the damage factor  D, a factor that is a link between the usual stress tensor and the effective stress:

(9) $$\sigma =\left(1-D\right)\tilde{\sigma }$$

where σ is the usual stress tensor, $\tilde{\sigma }$ is the effective stress, and D is the damage factor. The relationships among the tensors of the elastic modulus, elastic strain, autogenous shrinkage strain, thermal strain, and total strain are as follows.

(10) $$\dot{\tilde{\sigma }}=E\left(\xi \right){\dot{\varepsilon }}_{e}=E\left(\xi \right)\left(\dot{\varepsilon }-{\dot{\varepsilon }}_{\mathrm{as}}-{\dot{\varepsilon }}_{\mathrm{th}}\right)$$

Here, Poisson’s ratio is assumed to be a constant, and the relationship between the elastic modulus and the degree of hydration is as follows [56]:

(11) $$E\left(\xi \right)=E^{\infty }{\bar{\xi }}^{\beta }$$

(12) $$\bar{\xi }=\langle (\xi -\xi_{0})/{\left(\xi_{\infty }-\xi_{0}\right)\rangle }_{+}$$

where $\xi_{0}$ is the critical value of the degree of hydration, which is closely related to the hardening time of mortar. In fact, $\xi_{0}$ depends on the composition of the fine aggregate in mortar, the variety of cement, and the water-cement ratio [57], and it usually takes $\xi_{0}$ as a constant value of 0.1, referring to De Schutter and Taerwe’s research [58]. The parameter $E^{\infty }$ is the ultimate elastic modulus when the hydration is competed, and β is the material parameter, which can be obtained by fitting the experimental data.

Similar to Young’s modulus, the compressive strength can also be defined as a function of the degree of hydration [58]:

(13) $$f_{c}\left(\xi \right)=f_{c}^{\infty }{\bar{\xi }}^{\gamma }$$

where $f_{c}^{\infty }$ is the ultimate compressive strength when the hydration is competed, and γ is the material parameter that can be obtained from the experiment.

The damage factor D can be calculated from the elastic equivalent compressive strain seen as Equations (14) and (15) [59] and the code for the design of concrete/mortar structures (Chinese National Standard GB 50010-2010). This means that the concrete/mortar structure is perfect when D=0; the concrete/mortar structure is completely damaged if D=1. In this paper, the evolution of the damage factor is modified via coupling with the degree of hydration. The form is shown in Equation (16). Equations (17)–(20) are also transformed into the forms associated with the evolution of the degree of hydration:

(14) $$\hat{\varepsilon }=\sqrt{\langle \varepsilon_{\mathrm{el}}\rangle {}_{+}:\langle \varepsilon_{\mathrm{el}}\rangle {}_{+}}$$

(15) $$x\left(\xi \right)=\hat{\varepsilon }/\varepsilon_{c,r}\left(\xi \right)$$

(16) $$D\left(\xi \right)=\{\begin{array}{cc} 1-\left(\rho_{c}\left(\xi \right)\cdot n\left(\xi \right)\right)/\left(n\left(\xi \right)-1+x{\left(\xi \right)}^{n\left(\xi \right)}\right) & x\left(\xi \right)⩽1\\ 1-\rho_{c}\left(\xi \right)/\left(\alpha_{c}{\left(x-1\right)}^{2}+x\left(\xi \right)\right) & x\left(\xi \right)>1 \end{array}$$

(17) $$\rho_{c}\left(\xi \right)=f_{c}\left(\xi \right)/E\left(\xi \right)\varepsilon_{c,r}\left(\xi \right)=f_{c}^{\infty }\cdot {\bar{\xi }}^{\gamma -\beta }/E^{\infty }\cdot \varepsilon_{c,r}\left(\xi \right)$$

(18) $$n\left(\xi \right)=E\left(\xi \right)\varepsilon_{c,r}\left(\xi \right)/\left(E\left(\xi \right)\varepsilon_{c,r}\left(\xi \right)-f_{c}\left(\xi \right)\right)$$

(19) $$\varepsilon_{c,r}\left(\xi \right)=\left(700+172\sqrt{f_{c}\left(\xi \right)}\right)\times 10^{-6}$$

(20) $$\alpha_{c}\left(\xi \right)=0.157f_{c}{\left(\xi \right)}^{0.785}-0.905$$

where $\alpha_{c}\left(\xi \right)$ is the parameter value of the downward section of the stress–strain curve of the concrete/mortar under uniaxial compression in the hydration process, $\varepsilon_{c,r}\left(\xi \right)$ is the peak compressive strain of the concrete/mortar in the hydration process, and the other parameters are the intermediate variables that are mentioned in the Chinese National Standard GB 50010-2010.

### 3.2. Random Pore Model

The pore system in cement-based materials consists of four types of pores, namely macro-pores due to inadequate compaction, macro-pores due to deliberately entrained air, capillary pores, and gel pores. Pores can also be divided into four grades: very harmful pores (of d > 0.2 μm), harmful pores (of d at 0.05~0.2 μm), less harmful pores (of d at 0.02–0.05 μm), and harmless pores (of d < 0.02 μm). Many properties of cement-based materials are directly or indirectly related to the pore structure [60,61]. Therefore, a random pore model is created by deleting some elements in the whole mortar randomly to model the real status in the mortar, which was based on the results of MIP test shown in Table 3. Because the weak hole was taken into consideration, the response to external loads is more realistic in random pore model.

J.P. Ollivier [62] observed the porosity of the ITZ and cement matrix and found that the porosity of the ITZ is 2.5 to 3 times that of the cement matrix. In this work, a random pore distribution is considered; that is, in a homogeneous model, some elements are randomly deleted as pores, and the proportion of porosity in the ITZ and cement matrix is set to 2.5. For simplifying the simulation, only the very harmful pores (d > 0.2 μm) are considered in the random pore model, that the influence of ink-bottles would also be weaken. 

By using the random pore model, the elastic modulus of the solid element needs to be adjusted. The elastic modulus of the porous materials such as the mortar can be simply concluded by the method of generalized mixture rule (GMR) [63], which has a strict mathematical derivation. The GMR can be expressed as Equations (21) and (22):

(21) $$E_{c}^{J}=\overset{N}{\underset{i=1}{\sum }}\left(V_{i}E_{i}^{J}\right)$$

(22) $$\overset{N}{\underset{i=1}{\sum }}V_{i}=1$$

where *E* is the elastic modulus, V is the volume fraction of the component, the subscript i represents the ith phase, and c represents the composite consisting of N phases. The effects of microstructures are expressed by a scaling fractal parameter J. For a two-phase composite, Equation (23) can be simplified as follows:

(23) $$E_{c}^{J}=\left(1-V_{s}\right)E_{s}^{J}+V_{s}E_{w}^{J}$$

where $E_{s}^{J}$ is the elastic modulus of the strong phase, while $E_{w}^{J}$ is the weak phase. The concrete/mortar is considered a special two-phase composite in which null strength pores are distributed. Equation (23) can be rewritten as follows:

(24) $$E_{c}/E_{s}={\left(1-p\right)}^{1/J}$$

### 3.3. Calculation Parameters

The heat capacity of the matrix can be estimated using the law of mixtures when giving the volume fractions and heat capacity values of the composite cement. Equation (25) shows the law:

(25) $$\{\begin{array}{c} c_{freshpaste}=c_{water}f_{water}+c_{cement}f_{cement}\\ c_{paste\left(\xi \right)}=c_{freshpaste}\left\{1-A\left[1-\exp\left(-B\xi \right)\right]\right\}\\ c_{matrix}=c_{paste}f_{paste}+c_{fineagg}f_{fineagg} \end{array}$$

where c means the heat capacity (per unit volume), and f means the volume fraction in mortar. The subscript represents each component of the mortar. It should be noted that the heat capacity of the paste is variable with the hydration process, so that $c_{freshpaste}$ is the heat capacity of unhydrated mixture of water and cementitious blend. A and B are the material constants; in Bentz’s study [64], these values were measured with the w/c varying between 0.3 and 0.5.

The thermal conductivity of the mortar can be calculated from the well-known Hashin–Shtrikman (H-S) bounds [65] because the mortar is made of water, cement powder, and fine aggregates. Equation (26) shows the estimation method for calculating the H-S lower $\left(\lambda_{l}\right)$ and upper $\left(\lambda_{u}\right)$ bounds for the thermal conductivity when measuring the thermal conductivities ($\lambda_{1}$ and $\lambda_{2}$) and the volume fractions ($f_{1}$ and $f_{2}$) of the phases [64] for $\lambda_{1}$ ≥ $\lambda_{2}$:

(26) $$\{\begin{array}{c} \lambda_{l}=\lambda_{1}+f_{2}/\left(1/\left(\lambda_{2}-\lambda_{1}\right)+f_{1}/\left(3\lambda_{1}\right)\right)\\ \lambda_{u}=\lambda_{2}+f_{1}/\left(1/\left(\lambda_{1}-\lambda_{2}\right)+f_{2}/\left(3\lambda_{2}\right)\right) \end{array}$$

The effective thermal conductivity of the composite $\left(\lambda_{\mathrm{hom}}\right)$ can then be determined from the average of the H-S bounds as Equation (13):

(27) $$\lambda_{\mathrm{hom}}=\left(\lambda_{l}+\lambda_{u}\right)/2$$

By using the approach above, the thermal conductivity of the mortar components are derived. Table 4 summarizes the parameter results of the hydrothermal model. The parameters of the matrix can be calculated using Equations (25)–(27).

## 4. Simulation Results and Discussion

### 4.1. Numerical Samples

**Figure 6:**
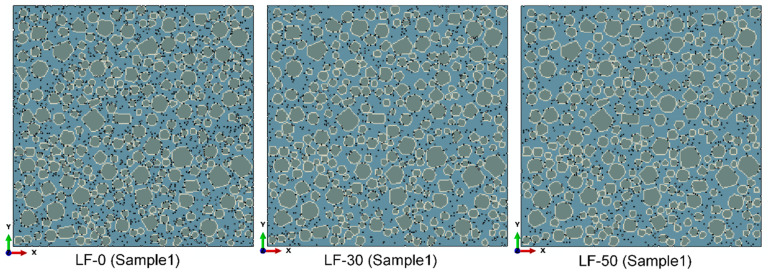
The numerical mortar specimens of LF-0, LF-30, and LF-50; the brilliant blue entity is the mortar matrix, the brown entities are the aggregate, the white entities are the ITZ, and the black vacancies are the pores.

Nine specimens with a size of 40 × 40 mm are constructed. The numerical models of LF-0, LF-30, and LF-50 are made of a mortar matrix, a fine aggregate, ITZ, and pores. For each kind of sample, three specimens that are different in aggregate and pore distribution are generated. One of the three specimens (Sample 1) is shown in Figure 6. The very harmful pores are realized by the random deletion of solid elements in the mortar matrix and ITZ. Here, it is assumed that the position of each component was fixed during the whole hydration process. The effect of fluidity that may lead to the position change of pore was not considered, because the random generation of the three sample can reduce the effect of fluidity. The number of deleted units in the numerical specimens is calculated according to the proportion of the very harmful holes (d > 0.2 μm) in the MIP test results. The proportion of the pore distribution between the cement matrix and ITZ is 2.5:1.

### 4.2. Model Validation

#### 4.2.1. Simulation of the “Hump-Type” Hydration Acceleration Stage

**Figure 7:**
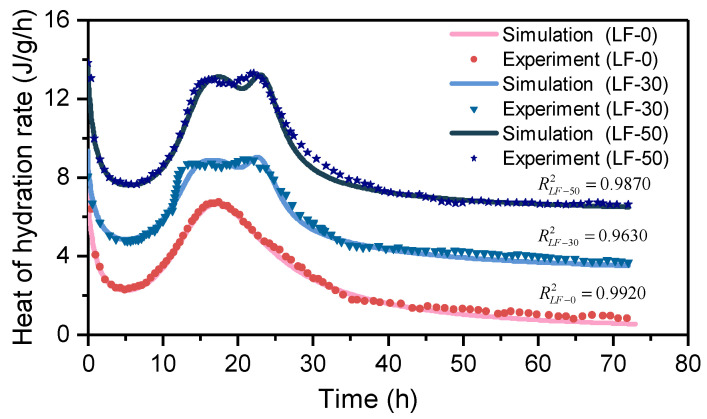
The evolution process simulation of the heat of hydration rate in LF-0, LF-30, and LF-50.

In this section, the cement pastes of samples LF-0, LF-30, and LF-50 are established to simulate the TAM test. The parameters of the cement paste in the hydration Equation (6) are calibrated, as shown in Table 5. The hydration process of the blend paste is then simulated using the chemical–thermal coupling method. The environment of the blend paste sample is set to adiabatic. Only an outlet of heat exists on the surface of the blend paste. The thermal conductivity of the outlet is very large. The heat of hydration rate can be calculated through the heat flow of the outlet. The boundary condition of the blend paste is set at a constant temperature of 25 °C, which is similar to the isothermal calorimeter. The heat of hydration rate curve is shown in Figure 7. For display purposes, the heat of hydration rate curve of LF-30 is shifted upward 3 J/g/h, and the LF-50 is shifted upward 6 J/g/h. For cement pastes of LF-0, there is no limestone powder inside, so there is only one peak in the hydration process. However, the second hydration acceleration stage is displayed in samples LF-30 and LF-50, which is caused by the limestone powder. From the simulation results, it can be seen that the curve of the numerical simulation is in good agreement with the experimental data, so the parameters can be applied to the mortar matrix and ITZ. The chemical–thermal model proposed in Equations (2) and (6) can continue to simulate the hydration process at long-term, because of the stable hydration of blend paste after 72 h.

#### 4.2.2. Elastic Modulus and Compressive Strength Evolution Simulation

**Figure 8:**
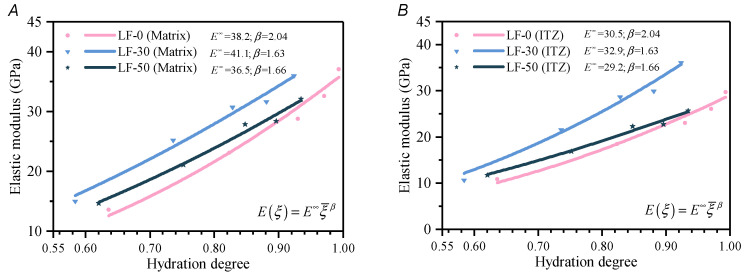

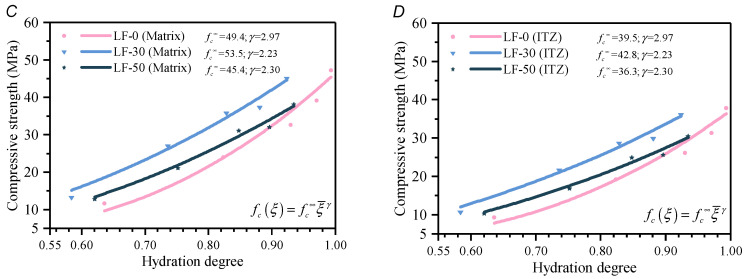
The elastic modulus and compressive strength evolution process in each phase of LF-0, LF-30, and LF-50. Elastic modulus evolution process: (**A**) matrix; (**B**) ITZ. Compressive strength evolution process: (**C**) matrix; (**D**) ITZ.

To obtain the relationship between the elastic modulus and compressive strength with the hydration degree in the numerical model, the elastic modulus of different ages is calculated by the method of the Chinese National Standard GB 50010-2010). Of course, other method mentioned in CEMHYD3D, HYMOSTRUC, etc., can also be used. The calculated elastic modulus is then matched with the degree hydration calculated by the chemical–thermal coupling method, and so is the compressive strength. The relationship above is developed by Equations (11) and (13). Figure 8 shows the elastic modulus and compressive strength evolution process of the matrix and the ITZ in LF-0, LF-30, and LF-50. Generally, the mechanical properties of the ITZ are 50–80% of the matrix, which is related to the bonding ability between the blend paste and aggregate [66,67]. The parameters of ITZ is reduced to 80% of that in the matrix [68,69], because the ITZ was only solid elements in the random pore model. It is worth noting that the elastic modulus parameters have been processed by a random pore model based on GMR theory, where J is 0.261, because the porosity of each component of the three mortars is within the appropriate range (porosity ≤ 0.3) [63].

### 4.3. Numerical Results and Discussion

#### 4.3.1. Compression Strength Simulation

**Figure 9:**
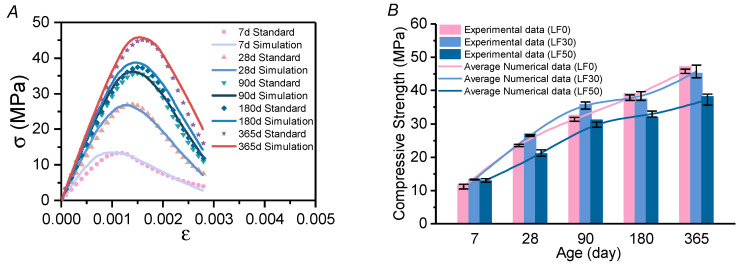
Strength–strain curves of the mortar specimens LF-30 at 7/28/90/180/365 days of age, and the comparison of the compressive strength between tested in physical experiments and the numerical simulation. (**A**) simulation of strength–strain curves at different days of age; (**B**) simulation of compressive strength at different days of age.

As described in Section 3.1, the evolution process of the hydration degree can be coupled to that of the mechanical parameters so that the elastic modulus of each component can be simulated. Based on the mechanical parameter studies in Section 4.2.2, numerical uniaxial compression tests of the mortar specimens at every test age are carried out. During the numerical test, the bottom surface of the mortar is fixed, and the top surface is loaded by displacement control at a loading speed of 0.0032 mm/s. The damage factor distribution and the stress field are simulated using the coupled chemical–thermal–mechanical model. Taking LF-30 as an example, the stress–strain curves are shown in Figure 9A. Each simulation curve is obtained by averaging the stress–strain curves of the mortar specimens with different aggregate distributions. In Figure 9A, the label “Standard” refers to the stress–strain curve calculated by the Chinese National Standard GB 50010-2010. The simulation curves fit well, so it can be concluded that the multi-field coupling method can accurately simulate the loading response under uniaxial compression. The comparison of the compressive strength between testing in physical experiments and the numerical simulation is shown in Figure 9B. The error bars are caused by the random distribution of pores using the random pore model. In addition, the simulated curves in Figure 9B are in good agreement with the experiment data at different ages of mortar, which proves that the coupled chemical–thermal–mechanical model can accurately simulate the mechanical properties over the entire evolution time.

In order to predict the strength of the mortar using the method of coupled chemical–thermal–mechanical method, it is advisable to use the same ternary Portland cement, so that the parameters in Table 5 are the same, which means that the aggregate gradation can be different from the mix proportion in Table 2. However, for new cementitious material with different compositions, the parameters should be recalibrated by TAM, MIP, and some mechanical testing.

#### 4.3.2. Failure Process under Uniaxial Compression

**Figure 10:**
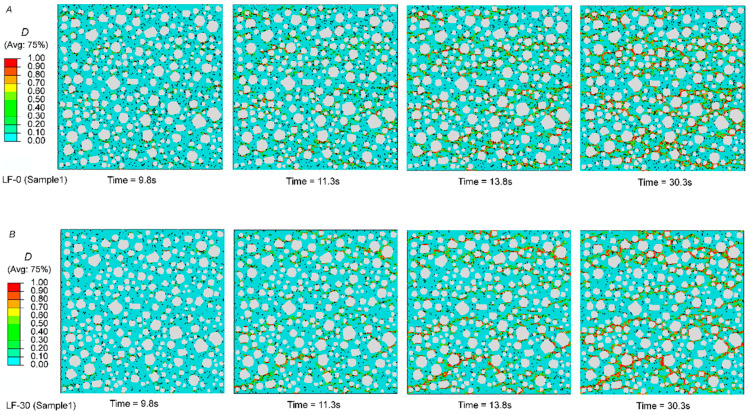

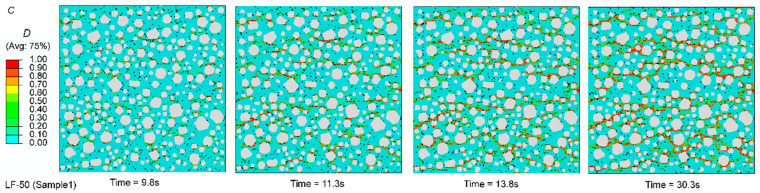
Evolution of the damage factor distribution of the mortar specimens LF-0, LF-30, and LF-50 at 28 days. (**A**) LF-0, (**B**) LF-30, (**C**) LF-50.

The damage factors can reflect the crack state. For the porous mortar specimen, the cracks first appear in the porous zone of ITZ, then develop along the porous zone of the matrix, and then gradually form complete cracks, which can be seen in Figure 10. The figure shows the evolution of damage factor distribution of the mortar specimens LF-0, LF-30, and LF-50 at the age of 28 days. After comparing the distribution of the damage factors of the three kinds of mixed mortar specimens, it can be proved that the more pores there are, the greater the probability of multiple cracks. This phenomenon can be explained by the filling effect of limestone powder [70]. Corresponding to the mortar specimen, the number of cracks in the mortar mixed with limestone powder is reduced because of the low porosity. This may be the reason for the high strength at early age.

## 5. Conclusions

This paper investigated the composite cement-based material with high amounts of fly ash and limestone powder by experimental tests and numerical simulation. The following conclusions have been drawn:

(1) In the OPC-FA system, limestone powder provides the nucleation sites that can accelerate the hydration effect at an early age, and 10%~20% of limestone powder is appropriate while excessive replacement may have a negative influence. Combined with fly ash, limestone powder has a filling effect, also there exitsthe aluminate phase reaction, which can both reduce harmful pores in the mortar and optimize the pore structure.
(2) In terms of cement-based materials, the coupled chemical–thermal–mechanical method can simulate the “hump-type” hydration stage of the composite mortar well, which is specifically caused by limestone powder. For the microstructure, the random pore model considers the pore differences between the ITZ and the mortar matrix, which can simulate the effect of harmful pores in the mortar.
(3) Combined with the coupled chemical–thermal–mechanical method, the random pore model can simulate and predict the hydration characteristics and mechanical properties of the mortar at different hydration ages, according to the information of aggregate grade, hydration rate, and porosity. The prediction property is in good agreement with the experimental results. The method can also be applied in other cement-based materials, while the fitting parameters should be recalibrated.

## Figures and Tables

**Table 1:** Chemical compositions of the ordinary Portland cement (OPC), fly ash (FA), and limestone powder (LP) (mass %).

Composition	SiO_2_	Al_2_O_3_	Fe_2_O_3_	CaO	SO_3_	Na_2_O	K_2_O	MgO	TiO_2_	LOI
OPC	22.75	7.92	4.03	55.60	2.82	0.19	0.86	2.09	0.33	3.16
LP	2.50	0.60	0.36	54.03	0.01	0.08	0.10	0.54	0.05	41.59
FA	53.41	25.79	4.28	2.60	0.30	0.42	1.38	2.79	—	3.67

LOI = loss on ignition.

**Table 2:** Mix proportion of the mortar mixture.

Sample	C (g)	LP (g)	LP (%)	FA (g)	FA (%)	S (g)	W (g)	Fluidity (mm)
OPC	450	0	0	0	0	1350	225	--
LF-0	225	0	0	225	50	1350	225	173
LF-10	225	45	10	180	40	1350	225	178
LF-20	225	90	20	135	30	1350	225	182
LF-30	225	135	30	90	20	1350	225	186
LF-40	225	180	40	45	10	1350	225	189
LF-50	225	225	50	0	0	1350	225	193

C = cement; LP = limestone powder; FA = fly ash; S = sand; W = water.

**Table 3:** Pore structure parameters of the mortar tested by MIP.

Sample	Porosity (by Volume)	Total Intruded Volume of Hg	Pore Distribution (by Volume) (%)			
			Diameter (d) of Pore (μm)			
	(%)	(mL·g^−1^)	<0.02	0.02–0.05	0.05–0.2	>0.2
LF-0	18.62	0.0901	47.38	27.24	7.77	17.61
LF-30	16.25	0.0785	48.05	35.74	3.43	12.78
LF-50	21.64	0.1052	48.79	33.35	9.99	7.87

**Table 4:** The basic parameters of the hydrothermal model.

Component	ρ (kg·m^−3^)	c (k·J·m^−3^·°C^−1^)	λ (J·h^−1^·m^−1^·K^−1^)
water	1000	4180	2160
cement	3180	2415	5580
aggregate	2681	2267	820
fly ash	2473	2190	4176
limestone powder	2760	2208	8800

**Table 5:** The parameters of the hydrothermal model.

Sample	$\beta_{1}$	$\beta_{2}$	$\beta_{3}$	$\bar{\eta }$	$\xi_{\infty }$	$\xi_{valley2}$	$\xi_{valley1}$	a	b	$Q_{\infty }$	EaR
	10^7^ h^−1^	-	-	-	-	-	-	-	-	(J·g^−1^)	(K)
LF-0	9.0	0.0059	−0.105	13.32	1.0	—	—	—	—	244	5000
LF-30	11.0	0.0038	−0.085	15.20	1.0	0.14	0.37	0.105	14.5	254	5000
LF-50	14.5	0.0038	−0.100	15.50	1.0	0.12	0.40	0.105	14.5	259	5000
